# Knowledge, attitudes and practices on hypertension among patients in a district hospital

**DOI:** 10.4102/safp.v67i1.6094

**Published:** 2025-05-15

**Authors:** Eslah H.H. Ahmed, Olga M. Maphasha, Sunday O. Okeke

**Affiliations:** 1 Department of Family Medicine, Faculty of Health Sciences, University of Pretoria, Pretoria, South Africa; 2 Department of Family Medicine and Primary Health Care, Faculty of Health Sciences, Sefako Makgatho Health Sciences University, Pretoria, South Africa

**Keywords:** knowledge, attitude, practices, hypertension, district hospital, Tshwane, South Africa, socio-demographic factor

## Abstract

**Background:**

Hypertension is a major global public health issue, with effective management relying heavily on patient adherence to lifestyle changes and medication. Understanding demographic influences on these behaviours is vital for targeted intervention. This study assessed knowledge, attitudes, and practices related to hypertension among patients at a district hospital in Tshwane, South Africa.

**Methods:**

A descriptive cross-sectional study used a structured, piloted questionnaire adapted from previous studies with 283 participants at a Tshwane district hospital.

**Results:**

The mean knowledge score was 55.2%, with gaps in understanding normal blood pressure (BP) values (46.29%) and risk factors (18.02%). Attitudes were positive, with 97.6% endorsing regular BP checks and 93.3% supporting salt reduction. Practices were moderate, with 70% never missing medication and 58% regularly monitoring weight. Higher education correlated with better knowledge and attitudes (*p* < 0.001, *p* = 0.001, respectively). Non-smokers and non-drinkers exhibited better health practices (*p* < 0.001). Age negatively correlated with knowledge (*r* = –0.15, *p* = 0.010) and attitudes (*r* = –0.19, *p* = 0.002).

**Conclusion:**

While attitudes towards hypertension are generally positive, knowledge and practices remain suboptimal. Targeted educational interventions, tailored to diverse socio-demographic factors, are essential to enhancing adherence.

**Contribution:**

This study identified gaps in hypertension management in Tshwane, aiding in the development of more effective, patient-centred educational programmes.

## Introduction

Hypertension, or high blood pressure (BP), is a major global health issue affecting millions. According to the World Health Organization (WHO), an estimated 1.28 billion adults aged 30–79 years worldwide have hypertension, with about two-thirds living in low- and middle-income countries (LMICs), such as South Africa.^1^ The global prevalence of hypertension has been rising, with approximately 1.13bn people affected in 2015, and this number is projected to reach 1.56bn by 2025.^1,2^ Furthermore, hypertension is already a leading cause of morbidity and mortality worldwide,^1,3^ and if current trends continue, it could indeed become the leading cause of non-communicable disease deaths by 2025, accounting for about 13% of all deaths globally.^4,5^ According to the WHO, cardiovascular disease (CVD) is the leading cause of death globally, accounting for more than 17 million deaths each year.^1^

Hypertension has become a significant burden in low-income countries, particularly in South and East Asia, as well as sub-Saharan Africa, over the past three decades.^6,7^ While hypertension was traditionally associated with high-income societies, there has been a shift in recent years, with higher rates of hypertension now observed in LMICs, particularly in Africa.^8,9^ One major factor is the rapid urbanisation and lifestyle changes occurring in these regions. As low-income countries experience economic development and urbanisation, there is a shift towards sedentary lifestyles, unhealthy diets, and increased stress, which can contribute to the development of hypertension.^7^ In Africa, hypertension is a major public health concern, and it is estimated that between 10m and 20m people out of the approximately 650m population in sub-Saharan Africa may have hypertension.^10^ Hypertension is the leading cause of heart failure in Africa and is responsible for more than half of the deaths from stroke in the region.^11^ In addition, hypertension is a major contributor to kidney disease, which is also a growing health problem in Africa.^12^ It is a major public health concern in South Africa; according to the 2016 South African Demographic and Health Survey, 46% of women and 44% of men are reported to be hypertensive or taking antihypertensive medication.^13^According to the WHO’s 2023 hypertension profile for South Africa, the age-standardised prevalence of hypertension among adults aged 30–79 years was 45% in 2019.^14^

Epidemiological studies have consistently shown that urban populations have a higher prevalence of hypertension compared to rural populations. For example, a study conducted in Nigeria showed that the prevalence of hypertension was 24.1% in urban areas compared to 13.3% in rural areas.^9^ Similarly, a study in China showed that the prevalence of hypertension was 33.5% in urban areas compared to 28.4% in rural areas.^15^

Additionally, there may be limited access to healthcare and a lack of awareness about the risks of hypertension in low-income countries.^16,17^ This can result in inadequate screening, diagnosis, and management of hypertension, leading to higher prevalence and poorly controlled hypertension in these regions. In many LMICs, there may be limited access to information, inadequate education, language barriers, cultural norms, and other factors that contribute to poor awareness among the general population.^7,8,18^ This lack of awareness can have negative consequences on multiple levels, including health outcomes, economic development, social well-being, and more.

The study conducted by Tesfaye et al.^5^ highlights the importance of patient education and awareness in the management of hypertension. Healthcare providers should educate patients on the potential complications of uncontrolled hypertension and encourage them to seek regular medical care.^5^ Patients should be empowered to take an active role in their healthcare by monitoring their BP at home, following a healthy lifestyle, and taking prescribed medications as directed.^7^

In addition, healthcare providers should work to address any misconceptions or fears that patients may have about hypertension and its treatment. By increasing awareness and understanding of hypertension and its potential complications, patients are more likely to engage in health-seeking behaviours that can lead to better BP control and improved health outcomes.

The increasing burden of hypertension in low-income countries is concerning; addressing the rising burden of hypertension in these regions requires a comprehensive approach, including efforts to promote healthy lifestyles, improve access to healthcare, raise awareness about the risks of hypertension, and implement effective strategies for prevention, diagnosis, and management of hypertension.

There is limited research exploring the knowledge, attitudes, and practices (KAP) related to hypertension among hypertensive patients in South Africa. These factors are critical, as they significantly influence treatment adherence, lifestyle modifications, and overall management outcomes. This study aims to fill this gap by assessing the level of KAP regarding hypertension management among hypertensive patients at a district hospital in South Africa. Additionally, it seeks to examine the correlation between patients’ socio-demographic characteristics and their KAP outcomes. The findings will provide valuable insights into patient behaviour, enabling the development of targeted interventions to enhance hypertension management and mitigate the associated health burden.

## Research methods and design

### Study design and setting

This cross-sectional descriptive study was conducted at Pretoria West Hospital, located in the Tshwane district, Gauteng province, South Africa. The hospital serves a diverse patient population from various socio-economic backgrounds, making it an ideal setting for assessing KAP related to hypertension.

### Study population and sampling

The study population comprised all adult patients aged 18 years and older who visited the outpatient department at Pretoria West Hospital from October 2023 to January 2024. It included patients diagnosed with hypertension or who had received treatment in the last 6 months and agreed to participate in the study. Pregnant or breastfeeding patients were excluded from the study. The estimated average population from which the sample was drawn was approximately 2275 individuals.

The sample size was determined using the precision-based method, based on a population size of 2275 patients, a 95% confidence interval (CI), a 5% margin of error, and a population proportion of 30%. The estimated sample size was 283 participants. A convenience sampling method was used where the first 10 patients who met the inclusion and exclusion criteria were invited to participate. All hypertensive patients visiting the outpatient department were approached, and the study was explained to them, including the consent process. This interaction provided an opportunity to distribute study information and consent forms. Patients who were interested in participating voluntarily completed the consent forms to proceed with the study.

### Data collection

Data were collected using a structured questionnaire adapted from previous studies,^19,20^ which was divided into several sections: (1) Socio-demographic information: This section gathered data on age, gender, education level, marital status, employment status, and other relevant demographic factors. (2) Knowledge questionnaire: Questions in this section assessed participants’ knowledge about hypertension, including understanding normal BP readings, identifying high BP, recognising its risk factors, symptoms, complications, and treatment options. (3) Attitude questionnaire: This part measured the participants’ attitudes towards hypertension, including their perceptions of the severity of the condition, beliefs about medication, and perceived benefits and barriers to treatment. (4) Practice questionnaire: Participants were asked about their health practices related to hypertension management, such as medication adherence, lifestyle changes, and frequency of medical consultations.

The questionnaire was designed to be comprehensive yet easy to understand, ensuring that all participants could provide accurate and honest responses. It was pilot-tested prior to the study to identify and rectify any issues related to clarity and reliability. The questionnaire was pilot-tested in 15 face-to-face interactions to evaluate the clarity, flow, and sequence of the questions, as well as to determine the average time required for participants to complete each question. This pilot testing allowed the research team to refine any questions that participants found difficult to understand, thereby minimising ambiguity and reducing the likelihood of incorrect responses.

Participants were recruited by the researcher in the outpatient department, where patients who met the inclusion criteria were approached. The patients were informed about the study’s purpose, procedures, and the voluntary nature of their participation. Before enrolment, participants were asked to provide written informed consent, ensuring that they fully understood the study and agreed to participate willingly. The questionnaire was administered in a private setting to maintain confidentiality and encourage honest responses. Participants had the option to complete the questionnaire themselves or receive assistance from a trained research assistant if needed. Once completed, the questionnaires were collected and reviewed for completeness and accuracy. The data were then entered into a Microsoft (MS) Excel spreadsheet, coded and prepared for analysis with the assistance of the statistician.

### Data analysis

The collected data were entered into a MS Excel spreadsheet, then coded and imported into the statistical software (IBM^®^ SPSS^®^ Statistics version 30) for analysis. The descriptive statistics show the participants’ socio-demographic profiles as well as their knowledge, attitudes, and practices in relation to hypertension. These are reported in frequencies and percentages (for categorical variables) and in means with standard deviation (s.d.) (for numeric variables). For each participant, the overall knowledge score was computed as a percentage of correct answers out of the total number of knowledge assessment items in the questionnaire. Therefore, the lowest possible score for a participant is 0, and the highest possible is 100. The overall attitudes score was computed based on the eight items in the 5-point Likert scale questions, thus giving the lowest possible attitudes score of 8 and a maximum possible score of 40 points. The overall practices score was similarly computed from the 8 practices-related items in the questionnaire, and therefore the lowest possible score was also 8 and the highest possible 40 points. Differences in the KAP scores based on the participants’ socio-demographics were examined using one-way analysis of variance (ANOVA). Correlations between KAP scores and numeric variables were examined using Pearson’s correlation coefficient. Multiple linear regression models were fitted to the data to identify factors that influenced the knowledge, attitudes, and practices in relation to hypertension. A significance level of 0.05 was used in the analysis.

The study assessed reliability by applying Cronbach’s alpha to test the internal consistency of the constructs in the survey questionnaire. A Cronbach’s alpha value of 0.7 or above indicated that the questionnaire had internal consistency and could effectively measure the study’s objectives as intended. A Cronbach’s alpha value of 0.7 or above indicated that the questionnaire had internal consistency and could effectively measure the study’s objectives as intended. The attitudes scale demonstrated strong reliability (α = 0.87). However, the practices scale initially had a lower reliability (α = 0.57), which improved to 0.67 when three negative practice items were excluded. While this rounds to 0.70, it is slightly below the conventional threshold. A combined alpha for attitudes and practices was also calculated, yielding 0.66 for all 16 items and 0.79 when excluding the three negative practice items. Given that the knowledge section consisted of yes/no and multiple-answer questions, Cronbach’s alpha was not applicable to this section. Therefore, the reliability of the full questionnaire was not reported as a single alpha value. Validity was ensured by using statistical parametric methods to confirm the precision of the results. To prevent bias, a pilot of the survey questionnaire was conducted, and adjustments were made based on the pilot results.

### Ethical considerations

Ethical clearance to conduct the study was obtained from the Faculty of Health Sciences Research Ethics Committee of the University of Pretoria (Protocol number 379/2023) and the provincial or Gauteng registration number of this study was obtained through Tshwane Research Committee (No. GP_202308_054) Furthermore, permission was requested and granted by the management of Pretoria West Hospital and Laudium Community Health centre to conduct research activities at the facility. Participant’s confidentiality and anonymity were guaranteed throughout the study and data collection process.

The names of the participants were not mentioned anywhere in the study. On the data collection tool, each participant was assigned a code that had no connection to his or her name. The collected data were stored electronically in a secure location and protected with a password, thereby maintaining strict confidentiality and anonymity throughout the study. Informed consent was obtained from participants to ensure voluntary participation and to confirm that no harm would come to them.

## Results

A total of 283 patients participated in the study. The socio-demographic characteristics of the participants summarised in Table 1 revealed a predominantly middle-aged to elderly population, with 50.88% aged 41–60 years and 37.46% over 60 years. The sample showed females constituted a larger proportion of the sample (60.42%). Most participants were married (52.65%), while 19.79% were single. Education levels varied, with 37.46% having secondary education and a combined 54.06% having completed matric, college, or university education. Regarding hypertension, 60.43% had been diagnosed within the last 1–10 years, with 23.67% having had the condition for 11–20 years. The mean duration of hypertension among participants was 11.65 years. A significant portion of the participants did not smoke (75.62%) or consume alcohol (74.20%).

**TABLE 1:** Socio-demographic factors of the participants (*N* = 283).

Variables	Descriptions	*n*	%
Age group (years)	21–40	33	11.66
	41–60	144	50.88
	> 61	106	37.46
Sex	Male	112	39.58
	Female	171	60.42
Marital status	Single	56	19.79
	Married	149	52.65
	Divorced	39	13.78
	Widowed	37	13.07
	Separated	2	0.71
Education level	No formal schooling	6	2.12
	Primary education	18	6.36
	Secondary education	106	37.46
	Matric certificate	75	26.50
	College education	44	15.55
	University education	34	12.01
Duration of hypertension since diagnosis (years)	1–10	171	60.43
	11–20	67	23.67
	21–30	33	11.66
	> 31	12	4.24
Smoking	Yes	69	24.38
	No	214	75.62
Alcohol intake	Yes	73	25.80
	No	210	74.20

The study revealed significant gaps in participants’ knowledge and awareness regarding hypertension (Table 2). Only 46.29% of the participants knew the normal BP reading, and a striking 72.79% were unaware of what constitutes high BP. While stress was recognised by 64.66% as a risk factor, only 18.02% identified all provided risk factors. Symptoms such as headaches and dizziness were known to 50.18% and 36.04% of participants, respectively, yet 27.92% correctly acknowledged all symptoms. Drug therapy was the most commonly known method of controlling hypertension (52%), but only 43% recognised that a combination of methods could be effective. Additionally, 71% understood the need for lifelong antihypertensive medication, while stroke (73%) and heart failure (60%) were the most recognised complications of uncontrolled hypertension. The overall mean knowledge score was 55.2% (95% CI: 52.6–57.9), with wide variability among participants.

**TABLE 2:** Participants’ understanding of hypertension.

Questions	Descriptions	*n*	%
Do you know the normal BP reading?	Yes	131	46.29
	No	152	53.71
	Total	283	100.00
Do you know what high BP is?	Yes	77	27.21
	No	206	72.79
	Total	283	100.00
What are the risk factors?	Stress	183	64.66
	Dizziness	34	12.01
	Family history of hypertension	144	50.88
	Excessive salt intake	82	28.98
	Excessive fat intake	105	37.10
	Being overweight	97	34.28
	All of the above	51	18.02
	Do not know	14	4.95
What are the symptoms present in hypertensive patients?	Headache	142	50.18
	Dizziness	102	36.04
	Fainting	11	3.88
	All of the above	79	27.92
	No symptoms	28	9.89
Methods used to control hypertension	Diet control	31	11.00
	Drug therapy	147	52.00
	Regular exercise	34	12.00
	All of the above	122	43.00
	Do not know	3	1.00
Do you have to take antihypertensive for life long?	Yes	201	71.00
	No	31	11.00
	Do not know	51	18.00
What are the complications of uncontrolled hypertension?	Heart failure	170	60.00
	Kidney failure	88	31.00
	Blindness	79	28.00
	Stroke	207	73.00
	Do not know	25	9.00

BP, blood pressure.

The study demonstrated a predominantly positive attitude towards hypertension management among participants. Most agreed with key preventive and management practices, such as avoiding added salt in the diet (93.3%), stopping smoking (69.7%), and stopping alcohol consumption (64.7%). Additionally, 93.0% supported regular exercise as a preventive measure, and 93.3% emphasised maintaining a healthy weight. Periodic BP checks were endorsed by 97.6%, while daily BP medications were supported by 97.2%. Furthermore, 95.4% agreed that patients with hypertension should avoid stress-inducing situations. The mean attitude score was 34.4 (95% CI: 33.9–34.9) out of 40, with an standard deviation (s.d.) of 4.3, indicating a generally favourable attitude (Table 3).

**TABLE 3:** Participants’ attitude towards hypertension (*N* = 283).

Statements	Agree		Uncertain		Disagree	
	*n*	%	*n*	%	*n*	%
It is good to avoid extra added salts in your diet.	264	91.0	3.0	1.0	16.0	6.0
Smoking cessation helps to prevent hypertension.	197	70.0	68.0	24.0	18.0	6.0
Stoppage of alcoholism helps to prevent hypertension.	183	65.0	89.0	31.0	11.0	3.0
Regular exercise helps to prevent hypertension.	263	93.0	17.0	6.0	3.0	1.0
Patients with high BP need to maintain their weight within normal limits.	264	93.0	17.0	6.0	2.0	0.7
BP should be checked periodically.	276	98.0	5.0	2.0	2.0	0.7
BP medication must be taken daily.	275	97.0	5.0	2.0	3.0	1.0
Patients with high BP should keep away from stress-inducing situations.	270	95.0	8.0	3.0	5.0	1.8

**Average**	**249**	**88.0**	**26.5**	**9.0**	**7.5**	**2.5**

BP, blood pressure.

The study revealed varying levels of engagement with practices related to hypertension prevention and management. A significant proportion of participants reported inconsistent monitoring of their BP, with 59% checking it sometimes, while only 14% never did so. Regarding salt intake, 54% always moderated their intake, though 27% never did. Fatty food avoidance was reported by 47% of participants as always being practised, with 32% never avoiding fatty foods. Alcohol consumption was more prevalent, with 78% reporting never consuming alcohol, while 5% always did. Regular physical exercise was practised by 36% always, though 34% never exercised. Monitoring body weight was done regularly by 58%, with 25% always checking it. Smoking was rare, with 76% never smoking, while 23% always smoking. Regarding BP medications, 70% never missed a dose, while 4% always missed a dose (Table 4). The mean practice score was 28.8 (95% CI: 28.2–29.4) out of 40, with an s.d. of 5.0, indicating moderate adherence to practices essential for hypertension.

**TABLE 4:** Participants’ practice towards hypertension.

Factors	Never		Sometimes		Always	
	*n*	%	*n*	%	*n*	%
How often do you measure your BP?	41.0	14.0	166.0	59.0	76.0	54.0
How often do you moderate your salt intake?	76.0	27.0	55.0	19.0	154.0	54.0
How often do you avoid fatty food?	90.0	32.0	59.0	21.0	134.0	47.0
How often do you consume alcohol?	221.0	78.0	49.0	17.0	13.0	5.0
How often do you perform the physical exercise?	95.0	34.0	86.0	30.0	102.0	36.0
How often do you check your body weight?	48.0	17.0	164.0	58.0	71.0	25.0
How often do you smoke?	214.0	76.0	3.0	1.0	65.0	23.0
How often do you miss the dose of your antihypertensive medications?	197.0	70.0	73.0	26.0	12.0	4.0

**Average**	**122.8**	**43.5**	**81.9**	**29.0**	**78.4**	**31.0**

BP, blood pressure.

In this study, the relationship between demographic factors and health-related KAP scores in a sample of participants, segmented by sex, marital status, highest education level, tobacco smoking, and alcohol consumption, is shown in Table 5. The data revealed no significant differences in the KAP scores based on gender (*p*-values: 0.641, 0.964, 0.816) or marital status (*p*-values: 0.936, 0.602, 0.665). However, a significant difference was observed in knowledge and attitudes based on the highest level of education (*p*-values: < 0.001, 0.001), with participants with higher education levels (college and university) having better knowledge and attitudes compared to those with lower education levels. The study also found that tobacco smoking had no significant effect on knowledge and attitudes but significantly influenced practices (*p* < 0.001), where non-smokers exhibited better health practices. Similarly, alcohol consumption did not significantly affect knowledge and attitudes (*p*-values: 0.467, 0.305), but it had a significant impact on health practices (*p* < 0.001), with non-drinkers showing more favourable health practices (Table 5).

**TABLE 5:** Knowledge, attitudes, and practices scores by socio-demographic factors (*N* = 283).

Variable	Scores								
	Knowledge			Attitude			Practices		
	*n*	Mean	s.d.	*n*	Mean	s.d.	*n*	Mean	s.d.
**Highest education level** †									
No formal schooling	6.0	43.9	13.2	6.0	32.8	3.7	6.0	26.7	3.9
Primary education	18.0	48.5	24.5	18.0	33.1	4.6	18.0	28.4	6.3
Secondary education	106.0	49.8	20.6	106.0	33.5	4.5	106.0	28.1	4.6
Matric certificate	75.0	51.7	20.8	75.0	34.4	4.0	75.0	29.3	4.5
College education	44.0	64.8	21.1	44.0	34.8	4.2	44.0	29.7	6.3
University education	34.0	73.1	20.5	34.0	37.1	3.6	34.0	29.1	4.9
**Tobacco smoking** ‡									
Yes	69.0	54.2	20.0	69.0	34.2	4.5	69.0	25.3	4.7
No	214.0	55.5	23.0	213.0	34.4	4.3	213.0	29.9	4.7
**Alcohol consumption** §									
Yes	73.0	56.9	22.4	73.0	34.8	4.3	73.0	26.7	5.0
No	210.0	54.7	22.4	210.0	34.2	4.4	210.0	29.5	4.9

s.d., standard deviation.

*, *p*-value statistically significant.

† , Knowledge *p*-value: < 0.001*, Attitude *p*-value: 0.001*, Practices *p*-value: 0.362;

‡ , Knowledge *p*-value: 0.674, Attitude *p*-value: 0.769, Practices *p*-value: < 0.001*;

§ , Knowledge *p*-value: 0.467, Attitude *p*-value: 0.305, Practices *p*-value: < 0.001*.

The study examined the correlations between age, duration of hypertension, tobacco smoking, alcohol consumption, and health-related KAP scores. Age was negatively correlated with knowledge (*r* = −0.15, *p* = 0.010) and attitudes (*r* = −0.19, *p* = 0.002), indicating that older individuals had lower knowledge and attitudes scores. However, age was not significantly associated with practices (*p* = 0.310). The duration of hypertension showed no significant correlation with any of the KAP scores (*p* > 0.05). Similarly, tobacco smoking duration and the number of cigarettes smoked per day were not significantly correlated with KAP scores (*p* > 0.05). Alcohol consumption duration had a significant negative correlation with knowledge (*r* = −0.25, *p* = 0.037), suggesting that longer alcohol use was associated with lower knowledge scores. Alcohol consumption frequency exhibited significant negative correlations with attitudes (*r* = −0.36, *p* = 0.002) and practices (*r* = −0.41, *p* < 0.001), indicating that more frequent alcohol consumption was linked to poorer attitudes and practices. These findings suggest that age and alcohol consumption patterns may play an important role in influencing health-related KAP (Table 6).

**TABLE 6:** Knowledge, attitudes, and practices scores by participants’ socio-demographic factors.

Factors	Knowledge score	Attitudes score	Practices score
**Age**			
*R*	−0.150	−0.190	0.060
*p*	0.010	0.002	0.310
**Duration of high BP (years)**			
*R*	0.030	−0.020	0.100
*p*	0.622	0.764	0.086
**Tobacco smoking duration (years)**			
*R*	−0.080	−0.080	−0.110
*p*	0.520	0.5110	0.390
**Number of cigarettes per day**			
*R*	−0.080	−0.030	−0.140
*p*	0.505	0.777	0.244
**Alcohol consumption duration (years)**			
*R*	−0.250	−0.190	−0.150
*p*	0.037	0.1050	0.196
**Alcohol consumption frequency (months)**			
*R*	−0.210	−0.360	−0.410
*p*	0.078	0.002	0.000

*R*, correlation coefficient; *p, p*-value; BP, blood pressure.

## Discussion

This study assessed the KAP of hypertensive patients at a district hospital, focusing on middle-aged and elderly individuals. A significant proportion of the participants (50.88%) were aged 41–60 years, and 37.46% were over 60 years. This finding aligns with previous studies attributing this rise to age-related vascular changes and lifestyle factors.^21,22^ However, participants aged 65 years and older were under-represented, possibly due to life expectancy limitations in the region. According to Statistics South Africa, the average life expectancy for Africans without human immunodeficiency virus or acquiredimmune deficiency syndrome is 65.1 years for males and 71.2 years for females.^23^

The study also revealed a gender disparity, with 60.42% of the participants being female. This finding is consistent with research conducted in Zambia and aligns with broader literature indicating that females are more likely to seek healthcare services.^20^ Post-menopausal females are also at higher risk of developing hypertension due to hormonal changes.^22^ Similar gender trends have been reported in studies conducted in Ghana, Zambia, and Egypt, highlighting that females are more likely to engage in healthcare and manage chronic conditions.^20,24,25^

Educational attainment among participants was relatively high, with more than half having completed matric or higher, which may contribute to better health literacy. However, significant knowledge gaps persisted. For example, only 46.29% of the participants could correctly identify normal BP levels. This reflects the persistent challenge of poor hypertension awareness in LMICs.^26^

The study highlighted participants’ understanding of hypertension risk factors, which were categorised into modifiable and non-modifiable factors. Modifiable factors such as obesity, physical inactivity, high salt intake, alcohol consumption, and smoking can be altered to reduce the risk of developing hypertension. Non-modifiable factors, including age, sex, genetic predisposition, and family history, are beyond individual control. Most participants identified stress, family history, and high fat intake as risk factors. However, only a minority recognised excessive salt intake, obesity, and ageing as important contributors to hypertension, consistent with the study conducted in Zambia.^20^

Participants demonstrated varying knowledge of hypertension symptoms. Headache was the most identified symptom, but few recognised that hypertension could be asymptomatic, a finding also noted in other studies.^20^ Regarding treatment, nearly three-quarters of the participants acknowledged the importance of lifelong antihypertensive medication, and many identified strokes as a major complication.

Smoking and alcohol consumption rates were low, with 75% of the participants reporting they had never smoked and 69% avoiding alcohol. These behaviours align with global trends among older adults managing chronic conditions.^27^ Younger participants were also more likely to avoid smoking and alcohol, reflecting positive health behaviours.

Salt reduction is widely recommended as an adjunct to pharmacological treatment to improve BP control.^28^ Reducing salt intake to 1.5 g/day can lower systolic BP by approximately 6 mmHg in hypertensive patients.^29^ Most participants in this study demonstrated an understanding of the importance of reducing salt intake, aligning with guidelines from the American College of Cardiology/American Heart Association.^29^

However, participants demonstrated poor adherence to physical exercise, a finding consistent with a cross-sectional study conducted in Nepal, which also reported low levels of exercise-related practices. These low levels are likely influenced by factors such as urbanisation and safety concerns.^19^ In this study, two-thirds of the participants monitored their BP occasionally, indicating a gap between knowledge and practice.

Despite these challenges, self-reported adherence to the medication was relatively high, with 70% of the participants reporting that they never missed a dose, a finding that contrasts with studies in Zambia, where poor adherence was observed.^20^ These positive attitudes align with studies showing a correlation between positive health attitudes and improved hypertension management.^30^ Nonetheless, further interventions are needed to support consistent adherence.^31^

This study highlights sub-optimal knowledge (55.2%), generally favourable attitudes and moderate adherence to hypertension management practice. Similar trends were observed in studies from South Africa and Zambia,^20,32^ with persistent knowledge gaps despite positive attitudes and varying adherence practice levels. While attitudes were slightly higher in this study and the Zambian study,^20^ inadequate knowledge and inconsistent adherence to practice remain key challenges, emphasising the need for targeted educational interventions.

Hypertension remains a significant public health concern globally, contributing to cardiovascular morbidity and mortality. Understanding patients’ KAP is essential for developing effective interventions to manage hypertension and prevent complications.

### Study limitations

The major limitation of this study is its cross-sectional design, which prevents causal inference, and reliance on self-reported data, introducing potential recall and social desirability bias. The study used convenience sampling, selecting the first available participants rather than randomly choosing from the entire population. This may lead to selection bias and limit the generalisability of the findings. Additionally, conducting the study in a single healthcare facility may limit the generalisability of findings to other regions with different socio-cultural and economic conditions.

### Recommendations

The study found that while 51% of the participants never missed a dose of their antihypertensive medication, 49% reported missing doses at least sometimes, highlighting the need for adherence strategies such as reminders and simplified regimens. Additionally, 59% of the participants only measured their BP occasionally, emphasising the importance of promoting regular BP screening through community health workers and clinics. Many participants struggled with lifestyle modifications, particularly moderating salt intake and avoiding fatty foods, indicating a need for enhanced dietary counselling and community-based health programmes. Education levels significantly influenced knowledge and attitudes towards hypertension, underscoring the importance of targeted health literacy initiatives tailored to different demographic groups. Addressing socio-demographic disparities, collaborating with community stakeholders, and conducting further research to assess intervention effectiveness are also crucial for improving hypertension management in this population.

## Conclusion

This study highlights important gaps in hypertension KAP, underscoring the need for targeted interventions to improve management. Participants displayed limited awareness of key aspects of hypertension, including normal BP levels, high BP, and associated risk factors. While there was general support for strategies like regular exercise and reducing salt intake, these habits were not consistently practised. Exercise, weight monitoring, and salt moderation require specific attention through tailored health education and community-based programmes that encourage healthier lifestyles.

Socio-demographic factors significantly influenced knowledge and behaviours, with differences observed across age and gender. Age affected awareness of smoking and alcohol cessation, as well as the importance of regular BP checks. Gender differences were evident in medication adherence, with females showing more consistent practices in taking BP medication.

To improve hypertension management, interventions must be customised to address these socio-demographic disparities and focus on promoting better lifestyle habits, such as exercise, weight management, and reducing salt intake. Collaborative efforts involving healthcare providers, community organisations, and public health programmes are essential to ensure more effective and equitable hypertension care. Further research will be necessary to evaluate the long-term impact of these strategies on hypertension outcomes.
